# The applicability of web-based solutions in headache epidemiology research

**DOI:** 10.1186/s10194-020-01141-2

**Published:** 2020-06-01

**Authors:** Kati Toom, Aire Raidvee, Mark Braschinsky

**Affiliations:** 1 Neurology Clinic, Tartu University Hospital, Tartu, Estonia; 2 Estonian Headache Society, Tartu, Estonia; 3 Institute of Psychology, University of Tartu, Tartu, Estonia

**Keywords:** Web-based survey, Online, Headache, Prevalence, Estonia

## Abstract

**Background:**

Epidemiological research of headache is vital but resource consuming prerequisite for evidence-based development in the field. Rapid evolution of information technology may provide new opportunities for population-based surveys. The aim of this study was to evaluate the applicability of web-based solutions in epidemiological studies of primary headaches.

**Methods:**

An online survey was conducted among 20–64 year old Estonian citizens, using a previously validated headache questionnaire. The participants were accessed through most popular portals and e-mail domains to get the maximum coverage of Estonian digital community. The resulting one-year headache prevalences were compared to those acquired in parallel from a population-based cross-sectional person-to-person study in Estonia.

**Results:**

Five thousand seven hundred eight entries were made by 5347 participants in the online study. Of the participants, 3896 (72.9%) had no headache, 1436 (26.8%) had only one and 15 (0.3%) had more than one type of headache. The study sample demographics were statistically significantly different from Estonian population and the prevalences were adjusted by age, gender, education and habitat. The proportion of headache sufferers was smaller in the online study sample (23.1% vs 41.0% in the population-based parallel person-to-person study). Among the headache sufferers the proportions of different headache diagnoses were similar across the two studies with the exceptions of episodic migraine and episodic tension-type headache. There were less migraine and more tension-type headache sufferers in the online study sample.

**Discussion:**

This is the first study addressing applicability of web-based solutions in headache related large epidemiological studies. Online approach presents a much faster means of data collection, larger samples, has mechanisms of avoiding data contamination and distinguishes the proportions of most primary headache disorders among the headache sufferers. However, the present online survey was significantly biased towards the people without headache, leading to underestimation of headache prevalence. This stems from the shortcomings related to method of sampling, access and engagement.

**Conclusion:**

Online headache epidemiology research could be a resource saving alternative to person-to-person studies, however, further research is needed to overcome the problems related to methods of sampling, access and engagement.

## Introduction

Epidemiological studies are important for acquiring information about disease patterns and aetiology as well as for creating the basis for the assessment of disease burden, cost and need for health services in society [1, 2]. However, large population-based epidemiological studies are usually resource and time consuming [2]. In the face of rapid digital evolution it would be beneficial to search for new methods for epidemiological surveys that could exploit the fast development of information technology. It certainly could be the case in headache epidemiology, bearing in mind that most primary headaches can be diagnosed based on history and do not require additional instrumental investigations. Nevertheless, online research poses several possible obstacles mainly concerning sampling-related biases. The latter probably demotivates researchers from testing online methodology in large-scale nation-wide studies. Therefore, in the context of epidemiological studies, the degree of biases involved in web-based methods have not been studied in an evidence-based, comparative manner.

Estonia is a North-Eastern European country with the population of 1.3 million [3]. It is one of the leading countries in the world regarding the usage of internet and web-based solutions per household – estimated at 86.2% among the population of 16–74-year-olds in 2016 [4]. In 2019 Estonia ranked 8th out of the 28 EU Member States in the European Commission Digital Economy and Society Index, showing that the use of internet services remains consistently high in this country [5]. This sets up potentially promising conditions for using e-technology in performing representative studies in headache epidemiology.

The aim of this study was to evaluate the applicability of a web-based approach in epidemiological studies of primary headaches by comparing the results of a web-based survey to a population-based epidemiological study in Estonia, the results of which have previously been published [6].

## Methods

### Surveys

Two parallel surveys were conducted, both from January 2016 to May 2017.

One of the surveys consisted of a population-based random sample of 2162 subjects who were interviewed by telephone or face-to-face using a previously validated questionnaire. The description of the methods and the results of this study have been previously published [6].

The other was a web-based survey. The participants included in the survey were Estonian citizens aged 20–64 and they were recruited via internet. For this purpose, an online recruitment campaign was performed. Advertisements for the same questionnaire were sent to different online portals and 150,000 e-mails were sent to six most popular e-mail domains in Estonia. The portals and e-mail addresses were chosen by an advertisement company and were aimed at maximum coverage of Estonian digital community. The advertisements and e-mails consisted of a short informative description of a health survey, avoiding any explanation that this was a headache survey in order to minimize participation bias. The advertisements and e-mails also contained a link to the headache questionnaire. The questionnaire was hosted by Tartu University Hospital’s server which provides a highly secure mode for participants’ data storage. In order to reach the questionnaire, the participants had to log in with their unique personal Estonian ID cards so that double entries could be traced and managed appropriately. This also secured that only Estonian citizens of the appropriate age were included, since the ID card data include the date of birth of the participants. At the end of the questionnaire there was a more thorough description of the purpose of the study explaining that it was a headache epidemiology study and making sure that participants, upon being fully informed, had the possibility of leaving the site without saving their data in case they decided not to give their consent. Otherwise, they saved their data by pushing the button „Finished“.

In order to encourage participation a lottery was announced on the advertisements and in the e-mails. The lottery draw was performed at the end of the study and two kinds of prizes were awarded to 11 random participants. The prizes were 10 sports-club memberships and 1 tablet-computer.

As multiple entries by single participants were expected, the following protocol was developed in order to manage these. In case the results of multiple questionnaire entries were identical, only the first entry was retained in the study. Thus, the multiple entries of the participants made by mistake or to enhance their chances of winning a prize by filling in the questionnaire several times were eliminated. If the results of the questionnaire did not overlap, the following 4 options were possible.

- Firstly, if the age reported by the participant did not match the age by ID (Estonian ID includes the date of birth) the entries were excluded as the participant was filling the questionnaire in under a false identity.
- Secondly, if one entry resulted in a headache diagnosis and another in no headache, the “headache” entry was accepted and “no headache” entries were excluded, because it is most probable that the “no headache” entries were completed in order to enhance the chances of winning the prize.
- Thirdly, if different entries by a participant resulted in different headache diagnoses that did not exclude each other, they were all accepted as different headaches may occur in one person.
- The fourth option was the case when different entries by a participant resulted in different headache diagnoses that excluded one another – for example the participant had both diagnoses of a chronic and an episodic form of the same headache, or both the probable and definite diagnoses of the same headache. In these cases, the chronic form was accepted and the episodic omitted, or the definite diagnosis accepted and the probable omitted, respectively.

When multiple entries were included from the same participant, s/he was still counted as a single participant having multiple headache cases. In other words, the total number of participants in the sample did not increase, but the number of respective headache cases did.

### Questionnaire

We used a structured questionnaire in Estonian that was developed by our study group and had undergone the specificity and sensitivity, as well as positive and negative predictive values’ estimation for most primary headache disorders [7]. The same questionnaire was used in the aforementioned population-based epidemiological person-to-person study of primary headaches in Estonia [6]. In the online study, the questionnaire was self-administered similarly to the original validation process [7].

The questionnaire consisted of four parts: 1) demographic data, 2) the headache diagnostic questionnaire, 3) headache-related burden and associated factors, and 4) enquiry on socioeconomic status and willingness to pay for effective headache treatment. The results of the enquiries in the third and fourth parts will be published elsewhere.

At the beginning of the diagnostic section the respondent was asked a screening question for headaches: “During the last year, have you had repeated headaches that were not caused by an acute infection, medication side effects, medical procedures, or consumption of toxic substances including alcohol?” If the respondent answered “yes”, s/he was introduced to questions targeting different aspects of the person’s headache (localization, laterality, character, intensity, preceding and accompanying symptoms, duration, frequency, response to indomethacin, association with certain situations/activities, precipitating factors, drug consumption, and history of head trauma) [7].

The participants were required to complete all the questions of the demographic and diagnostic parts in order to finish the questionnaire, thus avoiding missing data.

After filling in the headache questionnaire, an ICHD-3 beta based diagnostic algorithm [6–8] was applied and the respondent received one of the following diagnoses: no headache, episodic or chronic migraine, episodic or chronic tension-type headache, one of trigeminal autonomic cephalalgias, one of other primary headaches except for primary thunderclap headache and external-pressure headache, or, in case the described headache did not meet the criteria of any of the aforementioned entities, the unidentified headache was diagnosed [6, 7]. The headache had to fit either definite or probable criteria of ICHD-3 beta to be considered as a case [8].

### Statistical analyses

The main outcome of the online study were the one-year prevalences of primary headache disorders in the study sample. These prevalences were compared to the one-year prevalences of primary headaches in Estonian population acquired from the population-based person-to-person study published earlier [6]. Statistical methods used in both studies were identical. Data analysis was performed using *R* [9]. Sample weights were calculated using ANES (Americal National Election Study [10]) raking algorithm implemented in *R* package *anesrake* [11] (a standard approach in situations where data need to be simultaneously weighted for multiple demographic criteria). Comparison of the sample proportions was conducted using two-sample test for equality of proportions (with continuity correction).

## Results

**Fig. 1:**
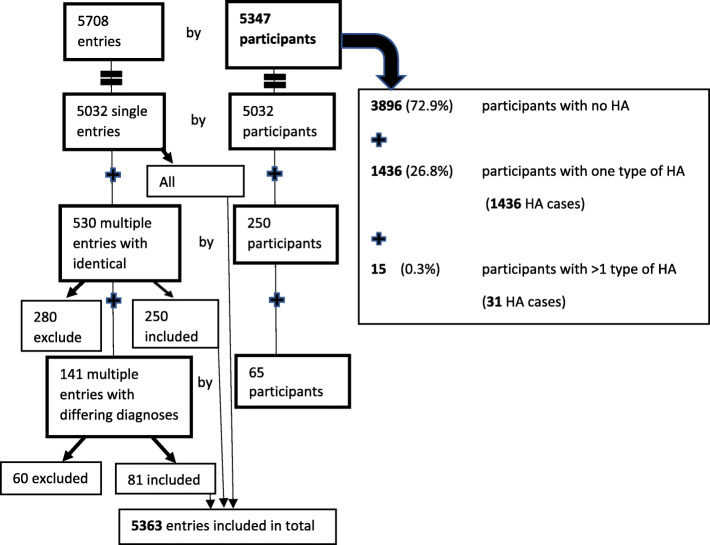
Study sample composition. HA – headache

During the period from January 2016 to May 2017, five thousand seven hundred and eight entries were made by 5347 individual participants. Five thousand and thirty two participants filled in the questionnaire only once, 250 participants made multiple entries which resulted in identical diagnoses, and 65 participants made multiple entries with differing diagnoses. After addressing the multiple entries according to the protocol, 5363 entries were included and 340 entries were excluded from the final analysis. Of the 5347 participants 3896 (72.9%) had no headache, 1436 (26.8%) had only one type of headache and 15 (0.3%) had more than one type of headache (Fig. 1).

The demographic data of the study sample are depicted in Table 1 alongside the data for Estonian population (data derived from Statistics Estonia, 01.01.2016 [12]).

**Table 1:** Comparison of Estonian population and survey sample

	General population, 20–64 years, 01.01.2016	Study sample	***p***-value
**Gender**, female (%)	50.4	71.5 (95% CI 70.3–72.1)	< 0.001
**Age** (%)			< 0.001
20–29 years	21.8	24.7	
30–39 years	23.1	31.0	
40–49 years	22.2	24.1	
50–59 years	22.1	15.4	
60–64 years	10.5	4.7	
**Marital status** (%)			< 0.001
Married	34.0	36.5 (95% CI 35.2–37.8)	
Not married (incl. Single, living with partner, divorced etc.)	66.0	63.5	
**Education** (%)			< 0.001
None, primary or basic	12.5	3.0	
Secondary or vocational	58.0	37.4	
Higher	29.5	59.6	
**Habitat** (%)			< 0.001
Urban	68.3	71.3	
Rural	31.7	28.7	

The study sample demographics was statistically significantly different from Estonian population. The proportion of women was higher, participants were younger, there were more married people, the education level and the proportion of people living in urban areas were higher in the study sample compared to the general population. Hence the study sample was adjusted to match the population demographically by weighting by age, gender, marital status, habitat and education.

The adjusted prevalences of primary headaches in the study sample (weighted by age, gender, marital status, habitat and education) are depicted in Table 2.

**Table 2:** Weighted one-year prevalences of primary headaches in the online study

PRIMARY HEADACHES	Number of cases in participating study sample	Number of cases after weighting	Weighted one-year prevalences (%) with 95% CIs
**All headache**	**1467**	**1234.7**	**23.1 (22.0–24.3)**
**All migraine**	**508**	**404.1**	**7.6 (6.9–8.3)**
● Episodic migraine	480	375.6	7.0 (6.4–7.8)
Definite	198	159.6	3.0 (2.6–3.5)
Probable	282	216.0	4.0 (3.5–4.6)
● Chronic migraine	28	28.5	0.5 (0.4–0.8)
Definite	12	12.7	0.2 (0.1–0.4)
Probable	16	15.8	0.3 (0.2–0.5)
**All tension-type headache**	**829**	**703.3**	**13.2 (12.3–14.1)**
● Episodic TTH	781	654.9	12.2 (11.4–13.2)
Definite	558	456.1	8.5 (7.8–9.3)
Probable	223	198.8	3.7 (3.2–4.3)
●Chronic TTH	48	48.4	0.9 (0.7–1.2)
Definite	33	30.2	0.6 (0.4–0.8)
Probable	15	18.2	0.3 (0.2–0.5)
**Trigeminal autonomic cephalalgias**	**2**	**5.3**	**0.1 (0.0004–0.2)**
**Other primary headaches**	**55**	**57.1**	**1.1 (0.8–1.4)**
**Chronic daily headache** (headache > 15 days a month)	**76**	**76.9**	**1.4 (1.1–1.8)**
**Unidentifiable**	**43**	**41.8**	**0.8 (0.6–1.1)**

*TTH* tension-type headache

The comparison between the adjusted prevalences of primary headaches in Estonian population-based person-to-person study sample [6] and in the online study sample (weighted by age, gender, marital status, habitat and education) is depicted in Table 3.

**Table 3:** Comparison of weighted one-year prevalences of primary headaches in Estonia [6] and in the online sample

PRIMARY HEADACHES	Weighted one-year prevalences (%) with 95% CIs in Estonian population aged 20–64	Weighted one-year prevalences (%) with 95% CIs in the online study sample
**All headache**	**41.0 (38.2–43.8)**	**23.1 (22.0–24.3)**
**All migraine**	**17.7 (15.7–20.0)**	**7.6 (6.9–8.3)**
● Episodic migraine	16.8 (14.8–19.1)	7.0 (6.4–7.8)
Definite	6.6 (5.3–8.2)	3.0 (2.6–3.5)
Probable	10.2 (8.6–12.1)	4.0 (3.5–4.6)
● Chronic migraine	0.9 (0.5–1.7)	0.5 (0.4–0.8)
Definite	0.7 (0.4–1.4)	0.2 (0.1–0.4)
Probable	0.2 (< 0.1–0.7)	0.3 (0.2–0.5)
**All tension-type headache**	**18.0 (15.9–20.3)**	**13.2 (12.3–14.1)**
● Episodic TTH	16.5 (14.4–18.7)	12.2 (11.4–13.2)
Definite	11.8 (10.1–13.8)	8.5 (7.8–9.3)
Probable	4.7 (3.6–6.0)	3.7 (3.2–4.3)
● Chronic TTH	1.5 (1.0–2.5)	0.9 (0.7–1.2)
Definite	1.5 (0.9–2.4)	0.6 (0.4–0.8)
Probable	0.1 (< 0.001–0.5)	0.3 (0.2–0.5)
**Trigeminal autonomic cephalalgias**	**0.4 (0.1–1.0)**	**0.1 (0.0004–0.2)**
**Other primary headaches**	**2.5 (1.7–3.5)**	**1.1 (0.8–1.4)**
**Chronic daily headache** (headache > 15 days a month)	**2.7 (1.9–3.8)**	**1.4 (1.1–1.8)**
**Unidentifiable**	**2.0 (1.3–3.0)**	**0.8 (0.6–1.1)**

*TTH* tension-type headache

**Fig. 2:**
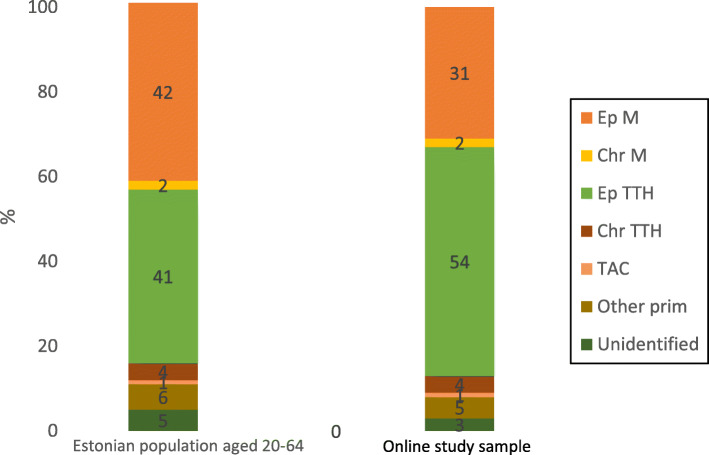
Proportions of different primary headache diagnoses among headache sufferers in the two samples. Ep M: episodic migraine; Chr M: chronic migraine; Ep TTH: episodic tension type headache; Chr TTH: chronic tension type headache; Other prim: other primary headaches; TAC: trigeminal autonomic cephalalgias

The percentage of headache sufferers in general was considerably smaller in the online study sample. However, among the participants who had headaches, the proportions of different headache diagnoses were similar in the two studies (Table 4 and Fig. 2) with only the proportions of episodic migraine and episodic tension-type headache being statistically different. There were proportionally less migraine and more tension-type headache sufferers in the online study sample compared to the population based person-to-person study sample in Estonia [6].

**Table 4:** Comparison of proportions of different primary headache diagnoses among headache sufferers in the two samples

Primary headaches	Weighted number of cases in Estonian population based person-to-person sample survey	Proportions of diagnoses among headache sufferers in the population based person-to-person sample (%)	Number of cases after weighting in the online sample	Proportions of diagnoses among headache sufferers in the online sample (%)	***p***-value for the proportions being different (χ2 tested)
Episodic migraine	204.6	42	375.6	31	< 0.001
Chronic migraine	11	2	28.5	2	1
Episodic TTH	200	41	654.9	54	< 0.001
Chronic TTH	18.7	4	48.4	4	0.96
TACs	4.5	1	5.3	1	0.41
Other primary headaches	29.8	6	57.1	5	0.31
Unidentifiable	24	5	41.8	3	0.21

*TTH* tension-type headache, *TACs* trigeminal autonomic cephalalgias

## Discussion

To the best of the authors’ knowledge, this study is the first one in the world to experimentally and evidentially address the question if web-based approach to the epidemiological studies of primary headache disorders is useful, and what pitfalls there may be expected. The comparison between the online and person-to-person survey methods is optimal, the most correct and informative only in case both surveys are performed within the same population during the same time period. Online solutions have been used in headache research previously [13–15] but there have been no attempts to conduct an online survey for primary headache epidemiology on such a large scale, involving a whole country.

One of the considerations in favour of online approach to epidemiological studies is time. Although the headache questionnaire was available for 15 months, we noticed that most of the entries were made in close temporal connection to the launches of the online advertisement and e-mail campaigns with most of the entries (*n* = 4082, 76% of total) made during 3 months’ time after the release of the campaign. This means that compared to the traditional methods of epidemiological studies it presents a much faster and cost-effective means of data collection. Additionally, the online study sample was considerably larger than the sample obtained in the offline person-to-person study that was carried out in parallel [6]. Hence the power of the study is also bigger and statistical corrections can be made with smaller error. This, we believe, is one of the biggest benefits of online surveys – the acquisition of large samples with less consumption of time and resource.

The prevalence of all headache in the study sample after adjusting it to the general population by age, gender, marital status, habitat and education was only 23.5% – almost 2 times smaller than the prevalence of all headache in the population-based random sample person-to-person survey performed in parallel [6]. This is likely to mean that the online survey was significantly biased towards the people without headache. We speculate that one of the reasons this could have occurred was the selection bias created by the lottery that was originally intended to enhance participation. Since the potential reward was a gym membership, it is possible that physically more active individuals may have been more likely to participate, reducing the prevalence of headaches [16]. It is also possible that a proportion of the participants did not take the trouble to fill in the questionnaire truthfully even if they had had a headache during the previous year and simply took the easy way out by saying they had not in order to be able to participate in the aforementioned lottery (we propose it could be called “convenience bias”). Although participants not admitting to having had a headache could not be totally excluded in the person-to-person survey, it is definitely more likely to have occurred in the web-based approach.

There is an evident imbalance between genders among the responders (71.5% were females). The gender differences in attending the internet in Estonia are not considerable, for example, 86% of women and 87% of men used the internet for sending and receiving e-mails in Estonia in 2017, whereas men used the internet more for reading the news and bank transactions than women (75% vs 70% and 63% vs 60% respectively) and social networks were attended slightly more by women than men (67% vs 63%) [17]. We believe that the imbalance of genders in this study might be explained by the tendency of women to be more concerned about health issues than men and therefore to attend health surveys more readily. However, since data in this study are adjusted for gender among other characteristics, this imbalance should not influence the prevalence rates considerably.

When omitting the participants without headache and looking at the proportions of primary headache diagnoses among those who reported headache in the online study, they are surprisingly similar to those found in the population based person-to-person random sample survey in Estonia [6] (Fig. 2, Table 4). The only statistically different proportions were those of episodic migraine and episodic tension-type headache, whereas the proportions of chronic migraine, chronic tension-type headache, trigeminal autonomic cephalalgias, other primary headaches and even unidentified headaches were almost identical. Furthermore, even the proportions of the statistically different episodic tension-type headache and episodic migraine are still similar to the proportions of their counterparts in the population-based person-to-person random sample study in the respect that these are still the largest and most prevalent diagnoses of primary headaches in the samples: in both studies, episodic tension-type headache and episodic migraine together comprise 83% and 85% of the primary headaches, respectively. The proportion of episodic tension-type headache is larger and the proportion of episodic migraine is equally smaller in the online study compared to the person-to-person study. One of the reasons for this discrepancy might be the fact that in the population based person-to-person study the questionnaire was administered face-to-face or by telephone interviews [6] whereas in the online study the questionnaire was completely self-administered. Since migraine diagnosis requires more detail (presence of accompanying symptoms etc) these nuances might be missed when the questionnaire is self-administered as opposed to the situation where the participant can ask clarifications from the interviewer. This is an important issue regarding the reliability of the future online studies and must be taken into account when designing these. On the other hand, most of the headache prevalence studies so far have demonstrated that tension-type headache usually is more prevalent than migraine in any given population [18, 19]. The prevalence of episodic tension-type headache in the population-based person-to-person random sample study was found to be 18.0% [6], which is exceptionally low when compared to other countries in the same North-Eastern European region. The reasons for this possible underestimation of episodic tension-type headache are discussed elsewhere [6]. However, this raises the question of whether the online study could have reflected the proportions of primary headache disorders in the population even more truthfully. Nevertheless, it is apparent that underestimation of headache prevalence would be one of the most troubling issues of online prevalence studies.

Another important factor, that our study underlined, is the necessity of having the participants identify themselves by some unique ID method. The 671 multiple entries by the same participants in the online study (about 12% of all the entries) certainly point to the fact that in such web-based surveys it is vital to have an identification method that would grant the means to manage the situation, especially where multiple entries increase the chances of winning a prize for participants. This again provides evidence that if lotteries and other similar “stimulating packages” are to be used to boost participation, it must be applied with utmost care to minimize the inevitable bias.

The main limitations of our study are related to the sampling methods. Valid conclusions of the population of interest (in our case Estonian population of 20–64 years of age) require probability sampling, where all members of the population have an initial probability of being selected to the study sample [2]. In our case it means that since almost about 87% of 16–74-year-olds in Estonia use internet on daily basis [4], about 13% of the population would be isolated from the possibility of being invited to a study when conducted online. However, there is no information about the age distribution of the non-users within this 13%. It is highly probable that most non-users are in the older age-group. Since our study sample’s upper age limit is 64 years, it is quite possible that the actual percentage of internet users within the targeted age-population is even higher than 87%, but this remains speculative due to lack of respective data.

We tested the hypothesis that high internet coverage among the general population in Estonia would be a factor sufficient enough for obtaining a representative sample by the chosen method of access and engagement. The analysis of the demographic data of the sample evidentially overruled this hypothesis. Our sample of 5347 participants was statistically significantly different form the general population of Estonia – the sample consisted of a younger, more educated and more urban group of people and there were more women than men among the participants. The smallest difference, although statistically significant, was in the marital status of the participants compared to the general population – there were more married people in the study sample. This shows that simply by addressing the digital community based on the most popular sites and domains does not grant a representative sample of general population even in the countries with highly developed information technology and in order to obtain representative samples in the future online epidemiological studies the methods of sampling, access and engagement must be more conservative [2]. There can be several possible solutions: the targeted invitation to the study could be linked to banking systems, e-health registries or e-mail addresses from national population registries in countries that use corresponding solutions extensively among adult population.

The evidence provided by our study should be considered when planning further research and generating guidelines for using web-based approaches in headache epidemiology.

## Conclusion

Our study shows that in the face of an already extensive and rapidly increasing usage of internet and IT-solutions among the general population, online headache epidemiology research could be a time- and resource efficient alternative in technologically developed countries. In addition to the possibility of obtaining larger study samples in relatively short time periods the IT solutions are capable of providing participant identification methods that enable avoiding data contamination. However, further research is needed to find more reliable methods of online access and engagement to gain representative samples and overcome the pitfalls of bias and most probably underestimation of headache prevalence.

## Data Availability

The dataset used and analysed during the current study is not publicly available due to the legal regulations of Data Protection Inspectorate of Estonia.

## References

[1] Vos T, Flaxman AD, Naghavi M (2012). Years lived with disability (YLDs) for 1160 sequelae of 289 diseases and injuries 1990-2010: a systematic analysis for the Global Burden of Disease Study 2010. Lancet..

[2] Stovner LJ, Al Jumah M, Birbeck GL (2014). The methodology of population surveys of headache prevalence, burden and cost: principles and recommendations from the global campaign against headache. J Headache Pain.

[3] Statistics Estonia. https://www.stat.ee/stat-population-at-beginning-of-year Accessed 19 Oct 2019

[4] International Telecommunication Union. Measuring the information society report 2017. Geneva, Switzerland 2017 Available from: itu.int/en/ITU-D/Statistics/Documents/publications/misr2017/MISR2017_Volume1.pdf Accessed 11 Jan 2020

[5] European Commission. Digital Economy and Society Index (DESI). 2019 Country report. Estonia https://eceuropaeu/digital-single-market/en/scoreboard/estonia Accessed 19 Oct 2019

[6] Toom K, Raidvee A, Allas KH (2019). The prevalence of primary headache disorders in the adult population of Estonia. Cephalalgia..

[7] Toom K, Laud T, Raidvee A, Braschinsky M (2016). Promising online tool for headache epidemiology: the PRILEVEL Pilot Study. J Neurol Neurosurg.

[8] Headache Classification Committee of the International Headache Society (IHS) (2013). The International Classification of Headache Disorders, 3rd edition (beta version). Cephalalgia.

[9] R Core Team (2019). R: A language and environment for statistical computing.

[10] DeBell M, Krosnick JA (2009) Computing weights for American national election study survey data. ANES Technical Report series, no. nes012427. In: Arbor A, Alto P. American National Election Studies. http://www.electionstudies.org/resources/papers/nes012427.pdf Accessed 24 Mar 2020

[11] Pasek J (2018). anesrake: ANES raking implementation.

[12] Statistics Estonia. https://www.stat.ee/population Accessed 1 Mar 2018

[13] Munjal S, Singh P, Reed ML (2020). Most bothersome symptom in persons with migraine: results from the migraine in America Symptoms and Treatment (MAST) study. Headache..

[14] Wilbrink LA, Weller CM, Cheung C (2013). Stepwise web-based questionnaires for diagnosing cluster headache: LUCA and QATCH. Cephalalgia..

[15] van der Ende-Kastelijn K, Oerlemans W, Goedegebuure S (2012). An online survey of exercise-related headaches among cyclists. Headache..

[16] Amin FM, Aristeidou S, Baraldi C (2018). The association between migraine and physical exercise. European Headache Federation School of Advanced Studies (EHF-SAS). J Headache Pain.

[17] Statistics Estonia. https://www.stat.ee/public/eurostat/Naiste-ja-meeste-elu-Euroopas/bloc-3c.html?lang=et Accessed 18 May 2020

[18] Stovner LJ, Andree C (2010). Prevalence of headache in Europe: a review for the Eurolight project. J Headache Pain.

[19] Stovner LJ, Hagen K, Jensen R (2007). The global burden of headache: a documentation of headache prevalence and disability worldwide. Cephalalgia..

